# Pachydermoperiostosis with bilateral ptosis and its associated systemic comorbidities: a rare case report

**DOI:** 10.11604/pamj.2023.45.88.38964

**Published:** 2023-06-20

**Authors:** Su Su Hlaing, Adeola Yvonne Field, Lizette Lillene Mowatt, Hsu Lei Yee, Cynthia Marie Noguera, Gordon Leon Palmer, Angela Christine Mattis, Christine Carol Nelson, James Christian Fleming

**Affiliations:** 1 Ophthalmology Division, Kingston Public Hospital, Kingston, Jamaica,; 2 Ophthalmology Division, Faculty of Medical Sciences, University of the West Indies, Kingston, Jamaica,; 3 University Hospital of the West Indies, Kingston, Jamaica,; 4 University of Tennessee Health Sciences Center, Memphis, Tennesse, United States of America,; 5 Kellogg Eye Center, University of Michigan, Ann Arbor, Michigan, United States of America,; 6 Hamilton Eye Institute, University of Tennessee, Memphis, Tennesse, United States of America

**Keywords:** Pachydermia, pachydermoperiostosis, ptosis, Jamaica, case report

## Abstract

Pachydermoperiostosis is a rare genetic disease known as primary or idiopathic hypertrophic osteoarthropathy (HOA)/Touraine-Solente-Gole syndrome. It is an autosomal dominant or recessive disorder comprising digital clubbing, periostosis, hyperhidrosis, and pachydermia (thickening of facial skin). Ocular manifestations are uncommon; however, blepharoptosis may occur. This case presented with severe bilateral ptosis due to the disease progression. A large 20 mm upper lid resection with levator advancement was performed to improve his ability to see. This is the first reported case of pachydermoperiostosis (PDP) in Jamaica. We present a rare case of pachydermoperiostosis with severe blepharoptosis, who attained a good result with surgical intervention.

## Introduction

Pachydermoperiostosis is a rare disease that has not previously been described in Jamaica or the Caribbean. This patient had all of the characteristics of the disease in its complete form, also known as primary hypertrophic osteoarthropathy [1]. The disease is often featured from a dermatologic and plastic surgery point of view. However, due to the ocular features examined, this is one of the few treated by ophthalmologists who removed the largest wedge resection reported. This led to a satisfactory patient clinical outcome. This case report demonstrates the successful treatment of severe ptosis in a patient with pachydermoperiostosis.

## Patient and observation

**Patient information and clinical history:** a 51-year-old male was referred to the Eye Clinic of Kingston Public Hospital, Jamaica, with a 15-year history of progressive bilateral ptosis obstructing his vision. He noted physical changes of wrinkled and thickened skin of his forehead from age 16, with progressive thickening of his eyelids from 25 years of age. He complained of swollen joints and hyperhidrosis of his hands and feet. Family history revealed all eight siblings were normal; however, his two paternal uncles have deep forehead furrows.

**Figure 1:**
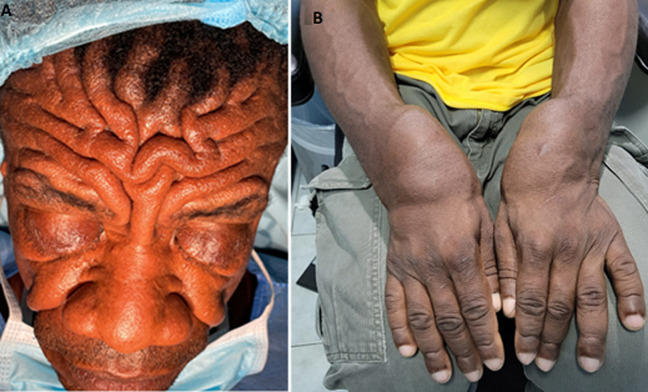
A) marked furrowing of the forehead and thickening of the skin; B) digital clubbing and swelling of the wrists and hands

**Clinical findings:** the patient had deep grooves in his forehead and severe bilateral ptosis (Figure 1 A). He also had digital clubbing and swollen joints involving the wrists (Figure 1 B), knees, and ankles bilaterally were present. He had papular lesions (sandpaper-like) on the anterior chest and mixed papular, pustules, and hypopigmented lesions on the back in a Y pattern.

Both lids were markedly thickened with severe bilateral ptosis. His palpebral fissures were 1 and 2 mm, and his marginal reflex distance (MRD) was +1 and 0 in the right and left eye, respectively. His upper lid excursion was 7 mm bilaterally. His visual acuity was 20/25 and 20/30 in the right and left eye, respectively.

**Figure 2:**
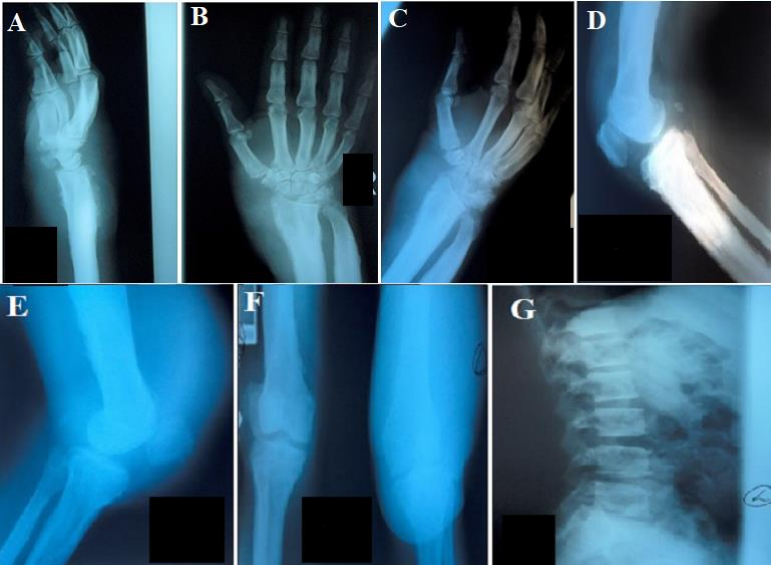
A, B, C) X-rays of both hands and wrists showing increased bone density and loss of corticomedullary differentiation, medullary expansion, and irregular periosteal reaction (spiculated), narrowing of joint spaces at radiocarpal joints associated with diffuse soft tissue swelling; D, E, F) X-rays of his knees showing spiculation around the patellae and proximal tibiae, with thickened cortex with medullary expansion; G) spinal X-ray showing increased bone density with mild degeneration of the spines with anterior osteophytes at L2-L5 and loss of lumbar lordosis

**Diagnostic assessment:** his blood investigations, including serum calcium and phosphate, were normal. X-rays of his hands and wrist showed increased bone density at the distal ulna and radius with loss of corticomedullary differentiation, medullary bone expansion, and irregular periosteal reaction. Spiculation was noted primarily at the distal ulna and radius. Joint space narrowing was also seen at the radiocarpal and midcarpal joints (Figure 2 (A,B,C)).

X-ray of his knees showed spiculated periosteal reaction around the patellae and proximal tibiae, thickened cortex, medullary expansion, and diffuse surrounding soft tissue swelling (Figure 2 (D,E,F)). Spinal X-rays showed increased bone density with mild degeneration to the lower lumbar spine (Figure 2G). His electrocardiogram was normal.

**Figure 3:**
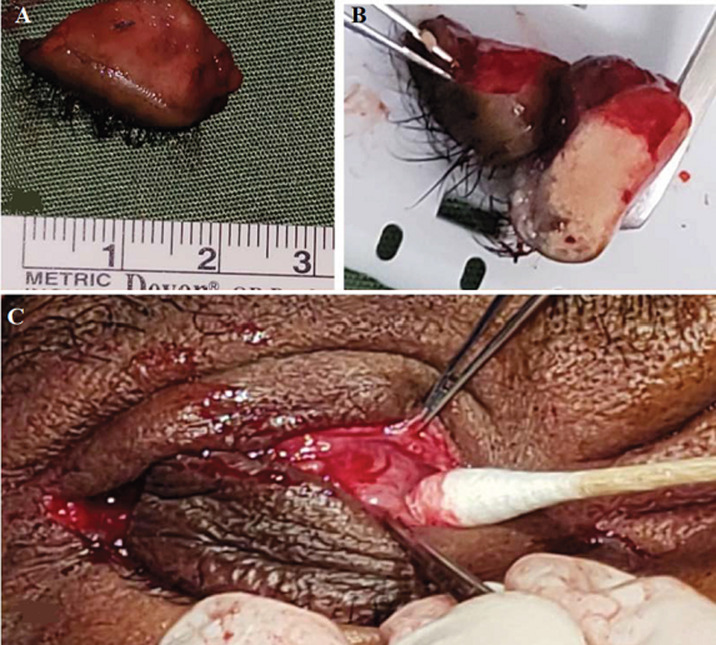
A) 20 mm full-thickness skin biopsy; B) cross-section of full-thickness right upper eyelid biopsy showing the thickened tarsal plate; C) right ptosis repair

**Diagnosis:** findings were consistent with pachydermoperiostosis with severe bilateral blepharoptosis.

**Figure 4:**
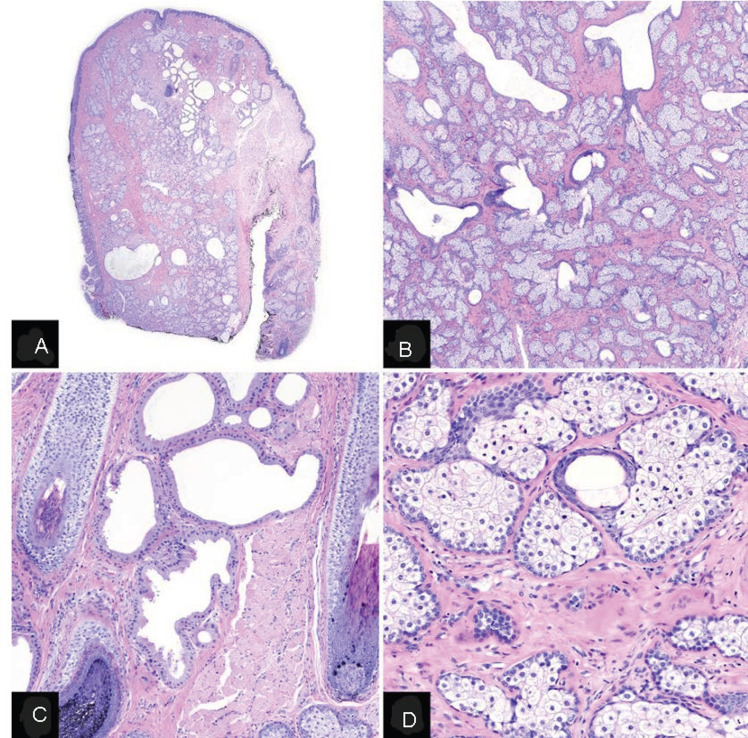
histopathological features A) low power; B,C) medium power; D) high power

**Therapeutic intervention:** he underwent right full-thickness wedge excision of the right upper eyelid (~ 20 mm with skin tissue biopsy) and levator muscle repair (Figure 3 (A,B,C)). Histology revealed sebaceous gland hyperplasia and prominent apocrine glands with mild hyalinization of the dermal collagen (Figure 4 (A,B,C,D)). The tarsal plate was almost four times the normal thickness.

**Figure 5:**
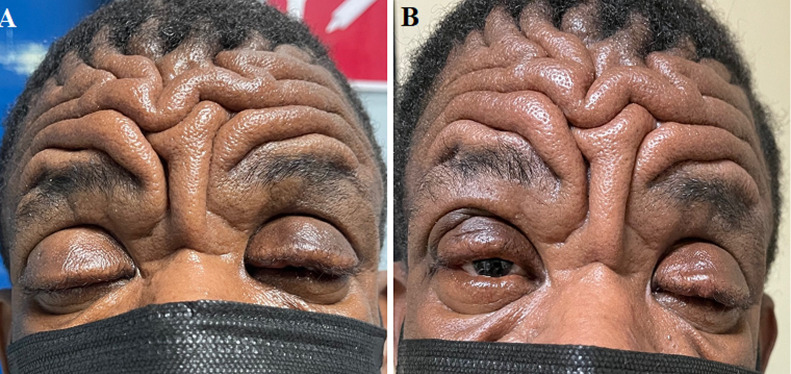
A) pre-ptosis repair; B) 2 weeks post right ptosis repair showing improvement of the right upper lid, allowing him to see through his visual axis

**Follow-up and outcome of intervention:** postoperatively, his right ptosis had improved, allowing him to see from his normal visual axis without an abnormal head posture (Figure 5).

**Patient perspective:** the patient was very happy with his improvement in vision and appearance. At his one-month postoperative visit, the patient stated, *"It is the best Christmas present to myself that I ever got, and I cannot wait to do the other eye. I can see much, much better now and much more"*.

**Informed consent:** written informed consent was obtained from the patient for this case publication.

## Discussion

Pachydermoperiostosis (PDP) is a rare genetic disorder with systemic findings of pachydermia (skin thickening) and periostosis (digital clubbing) commencing from adolescence. The prevalence is 0.16%, with male to female ratio of 7: 1 [2]. Although an autosomal dominant with incomplete penetrance and autosomal recessive inheritance have been proposed, an X-linked or testosterone-promoting proliferation may explain the higher incidence in males [3].

There are limited published ocular manifestations; blepharoptosis and floppy eyelid syndrome have been described [4]. The mechanical ptosis is secondary to tarsal plate hypertrophy and thickened periocular tissues, as seen in our biopsy. Visual loss has been reported in one case secondary to severe phlyctenular keratoconjunctivitis [5]. Our case had significant cutis verticis gyrate of his forehead compared to other published cases [6-8]. His clinical features were in keeping with primary hypertrophic osteoarthropathy with enlarged diaphysis, increased bone density, and periosteal proliferation on X-rays [7-9]. Our case had a spiculated appearance of his bones, not previously described. Bilateral transient patellar dislocation has been reported in the literature [ 10].

Gastrointestinal features, including hypertrophic gastric folds with multiple ulcers in the gastric antrum, may be present; a gastric mucosa biopsy reveals mild and chronic inflammation and metaplasia [8]. Myelofibrosis with anaemia has been reported [6]. Skin biopsy usually shows mucin deposition and hypertrophic sebaceous glands [8].

The inheritance pattern may be autosomal dominant and recessive; due to the SLCO2A1gene deficiency and 15-hydroxy-prostaglandin dehydrogenase (HPGD) gene mutations, respectively [8]. There are seven novel subtype mutations of the SLCO2A1 gene, which encodes a prostaglandin transporter responsible for the cellular uptake of prostaglandin E2 [11]. The HPGD gene encodes 15-hydroxyprostaglandin dehydrogenase, which catabolizes prostaglandin E2. The deficiency of either SLCO2A1 or HPGD gene mutation results in chronic prolonged elevated inflammatory markers of prostaglandin E2 levels, resulting in the overstimulation of osteoblasts, osteoclasts, and fibroblasts and clinical features of PDP [8].

Medical management includes palliative care for painful polyarthritis/osteoarthropathy with Non-steroidal anti-inflammatory drugs (NSAIDs), corticosteroids, pamidronate, tamoxifen citrate, and risedronate sodium [12]. Botulinum toxin type A injection has improved the cosmetic appearance of coarse facial features of PDP [12]. However, the Botox mechanism of action remains unknown, as the primary cause of deep wrinkles in PDP is thought to be secondary to the thickening of the dermis layer and not to the repetitious use of the frontalis and corrugator muscles. Surgical options include vagotomy for articular pain and swelling and correction for digital clubbing [12].

Hypertrophy of palpebral tissues causes thickening and lengthening of the upper eyelids, which override the lower eyelids [13]. Diffuse intratarsal ectopic lacrimal gland tissue has been described [14]. For visually significant blepharoptosis, surgery includes levator palpebrae superioris resection and full-thickness wedge resection of tarsal hypertrophic tissues. These patients may initially present to the ophthalmologist with ptosis; it is important to be aware of the other clinical findings. Our patient presented at an older age, with significant cutis verticis gyrate and severe ptosis compared to previously published cases.

Pachydermoperiostosis (PDP) is a rare genetic disorder with a wide clinical spectrum of presentation that requires a multidisciplinary approach (dermatology, orthopaedics, ophthalmology, gastroenterology) with a multisystem workup and pedigree evaluation.

## Conclusion

This is the first reported presentation of PDP in the Jamaican population and Caribbean region. His digital clubbing, joint deformities (wrists, knees, and ankles), pachydermia, cutis verticis gyrate, hyperhidrosis and radiological and pathological findings confirm the diagnosis. Despite the thickened tarsal plate, surgical intervention (lid wedge resection of the lid with the levator palpebral superioris advancement) resulted in a good functional and cosmetic result. Management of this rare condition should be multidisciplinary.
